# Physiotherapeutic Management for Acromioclavicular Joint Sprain With Volar Intercalated Segment Instability at the Wrist: A Case Report

**DOI:** 10.7759/cureus.58399

**Published:** 2024-04-16

**Authors:** Harsh R Nathani, Swapnil U Ramteke, Pratik R Jaiswal

**Affiliations:** 1 Sports Physiotherapy, Ravi Nair Physiotherapy College, Datta Meghe Institute of Higher Education & Research, Wardha, IND

**Keywords:** laser, rehabilitation, mulligan’s movement with mobilization, cryotherapy, acromioclavicular joint

## Abstract

Among sports enthusiasts and young individuals, acromioclavicular (AC) joint injuries are highly prevalent. In this, we discussed the comprehensive assessment and management of a 22-year-old male patient who is a student by occupation and a recreational badminton player who presented with left shoulder and wrist pain following a road traffic accident. The study highlights the clinical findings, diagnostic assessment, and therapeutic interventions for the patient with volar intercalated segment instability and a grade 1 AC joint sprain. The methodology involves a case report of the patient’s clinical evaluation, including range of motion, manual muscle testing, and diagnostic imaging. The patient was managed conservatively with physiotherapy interventions, including Mulligan’s movement with mobilization, cryotherapy, light amplification by stimulated emission of radiation, and progressive exercises. The results of the study demonstrate the successful implementation of a multidisciplinary conservative management approach for alleviating pain, restoring function, and promoting optimal recovery for the patient. The implications of the study underscore the significance of tailored physical therapy rehabilitation in the management of AC joint sprains and wrist instabilities.

## Introduction

Acromioclavicular (AC) joint injuries are prevalent among sports, and young people account for more than 40% of all shoulder injuries [1]. The AC joint is diarthrodial in nature and is reinforced by the AC ligament, which consists of an anterior, posterior, inferior, and superior portion. Two coracoclavicular ligaments and the coracoacromial ligament serve as supportive structures [2]. AC injuries are common during athletic events, motor vehicle collisions, bicycle falls, and other sporting activities (e.g., skiing). This injury is also seen in sports involving collisions, including football, lacrosse, and ice hockey [3]. The AC joint is most commonly damaged when there is trauma directly to the acromion process or the shoulder’s lateral aspect with the arm positioned in adduction. Patients often present with anterosuperior shoulder pain radiating to the neck or shoulder and may experience swelling, bruising, or deformity of the AC joint [4]. They may also experience a restricted range of motion due to pain. A “piano key sign” may be seen, with an elevation of the clavicle rebounding after inferior compression. Standard X-rays are adequate for diagnosing AC joint injuries, but additional views like the Zanca view, bilateral anteroposterior (AP) views, weighted stress views, and ultrasound or MRI may be considered for further diagnostic evaluation [5]. AC joint injuries are classified from type I to type VI, with type I and type II sprains requiring nonoperative management. Type III sprains are usually treated nonoperatively, but if the displacement is greater than 75%, then it requires surgical management [6]. Injuries from type IV to VI involve stabilization and reduction techniques; for acute injuries, hook plates, Bosworth screws, tension band wiring, and Endobutton are utilized [7]. Chronic AC joint disruptions involve debridement, ligament reconstruction, and stable fixation procedures, including modified Weaver-Dunn, Mazocca, Neviaser, Rockwood, Docking, and Mumford procedures [8].

Carpal instability and its biomechanics have evolved over time as radiography and arthroscopy have advanced. In terms of carpal stability, the lunate is crucial and functions as an intercalated segment; intercalated segment instability results from the lack of anchoring of this vital component [9]. Another less prevalent form of dissociative carpal instability is known as volar intercalated segment instability (VISI) [10]. VISI is an abnormality characterized by pathological volar flexion of the lunate, with or without a corresponding position of the remaining proximal row carpal bones [11]. Physical therapy rehabilitation is a nonoperative management program that is incorporated to address a range of motion, strength, and neuromuscular control [12]. Manual therapy techniques like mobilization with movement (MWM), which involves the application of a sustained glide at a peripheral joint as a manual therapy technique to address positional faults caused by injury or strain, are beneficial in reducing pain and improving patient outcomes [13]. By doing so, it aims to elicit a hypoalgesic effect and potentially trigger sympathoexcitation. Moreover, tailored rehabilitation with specific progressive exercises for wrist stabilizing musculature is also beneficial for dealing with wrist instabilities [14].

## Case presentation

Patient information

A 22-year-old male patient, a student by profession and a recreational badminton player, came to the hospital with complaints of pain in his left shoulder and left wrist with abrasion wounds soon after he met with a road traffic accident (RTA). He fell on an outstretched hand and landed on the palmar aspect of his hand. With the following complaints, the patient was advised to undergo a routine radiographic investigation for his left wrist and shoulder, which revealed carpal instability in the wrist and a grade 1 sprain of the AC joint in the shoulder radiograph. He was managed conservatively by the application of povidone-iodine for abrasion wounds, the injection of diclofenac for pain relief, and the injection of tetanus to prevent any chance of infection. As the patient was developing difficulty moving his wrist and shoulder, he was referred for physiotherapy for further management.

Clinical findings

After getting verbal consent from the patient, he was evaluated. The patient was seen in a sitting position and was conscious, cooperative, and well oriented to time, place, and person; he was right-hand dominant. The patient had pain over the anterior aspect of his left shoulder and dorsum of his wrist and was assessed on the Visual Analogue Scale, which was 4/10 on rest and 7/10 on activity. The pain was dull and aching in nature; the onset was sudden and gradually increased. The pain got aggravated by performing wrist flexion and extension and shoulder flexion, extension, and cross-arm adduction movements. There were not any temporal variations associated with it. On observation, the patient kept his left shoulder in protraction, adduction, and external rotation and his wrist in slight flexion. On inspection, there was slight swelling present over the dorsum of the left wrist. On palpation, the swollen tissue was slightly warm, and tenderness of grade 3 was present over the lateral aspect of the dorsum of the wrist and the anterior aspect of the shoulder joint. The quality of movement was grade 4, which is painful and incomplete. The active range of motion assessment for the patient is depicted in Table 1. Manual muscle testing for both wrist and elbow musculature is shown in Table 2.

**Table 1:** ROM assessment for the left wrist and shoulder in comparison to the unaffected extremity ROM, range of motion

Movements	ROM for left shoulder (affected)	ROM for right shoulder (unaffected)
Wrist flexion	0-40°	0-80°
Wrist extension	0-40°	0-70°
Radial deviation	0-10°	0-20°
Ulnar deviation	0-10°	0-30°
Pronation	0-50°	0-80°
Supination	0-60°	0-70°
Shoulder flexion	0-130°	0-180°
Shoulder extension	0-30°	0-60°
Shoulder abduction	0-120°	0-180°
Shoulder adduction	0-30°	0-50°
Shoulder internal rotation	0-40°	0-70°
Shoulder external rotation	0-60°	0-90°

**Table 2:** Manual muscle charting evaluation for the left wrist and shoulder

Muscles	Grading
Wrist flexors	2+/5
Wrist extensors	3-/5
Shoulder flexors	3+/5
Shoulder extensors	2+/5
Shoulder abductors	3/5
Shoulder adductors	2+/5
Shoulder internal rotators	3-/5
Shoulder external rotators	4-/5

Diagnostic assessment

**Figure 1:**
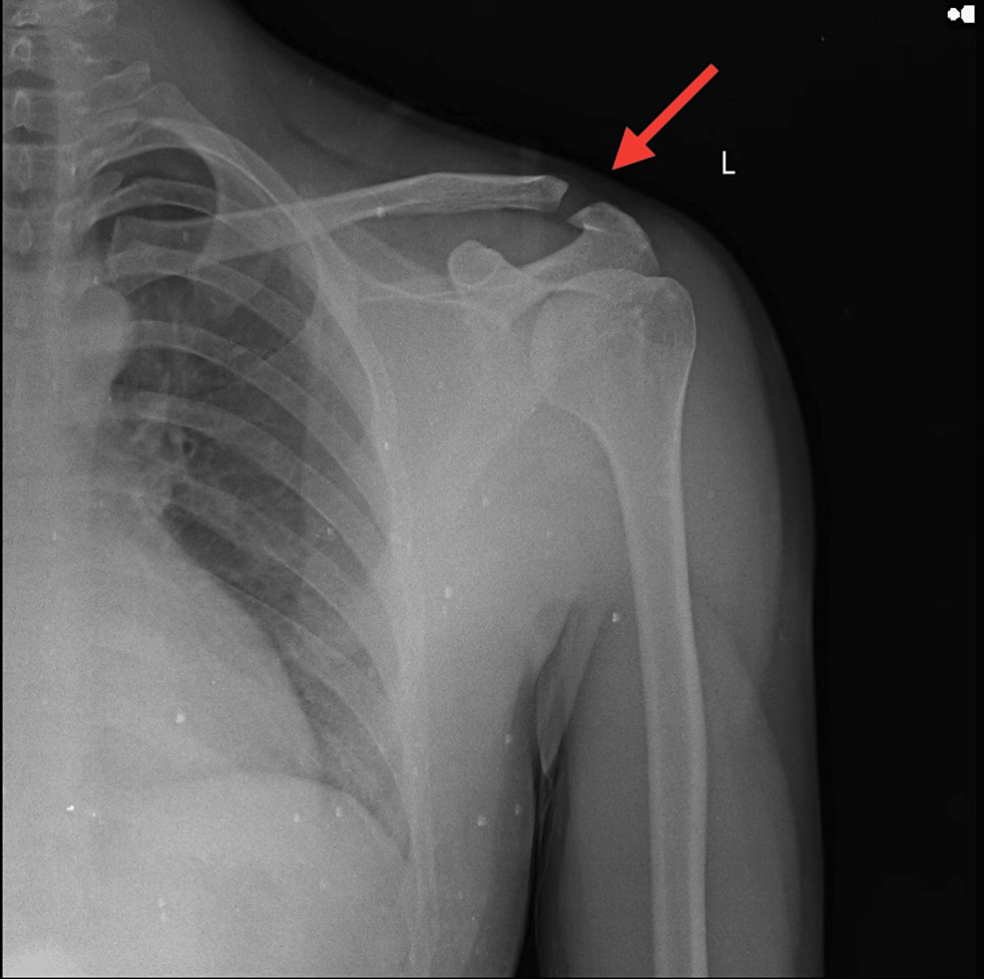
Radiograph of left shoulder showing grade 1 AC joint sprain AC, acromioclavicular

**Figure 2:**
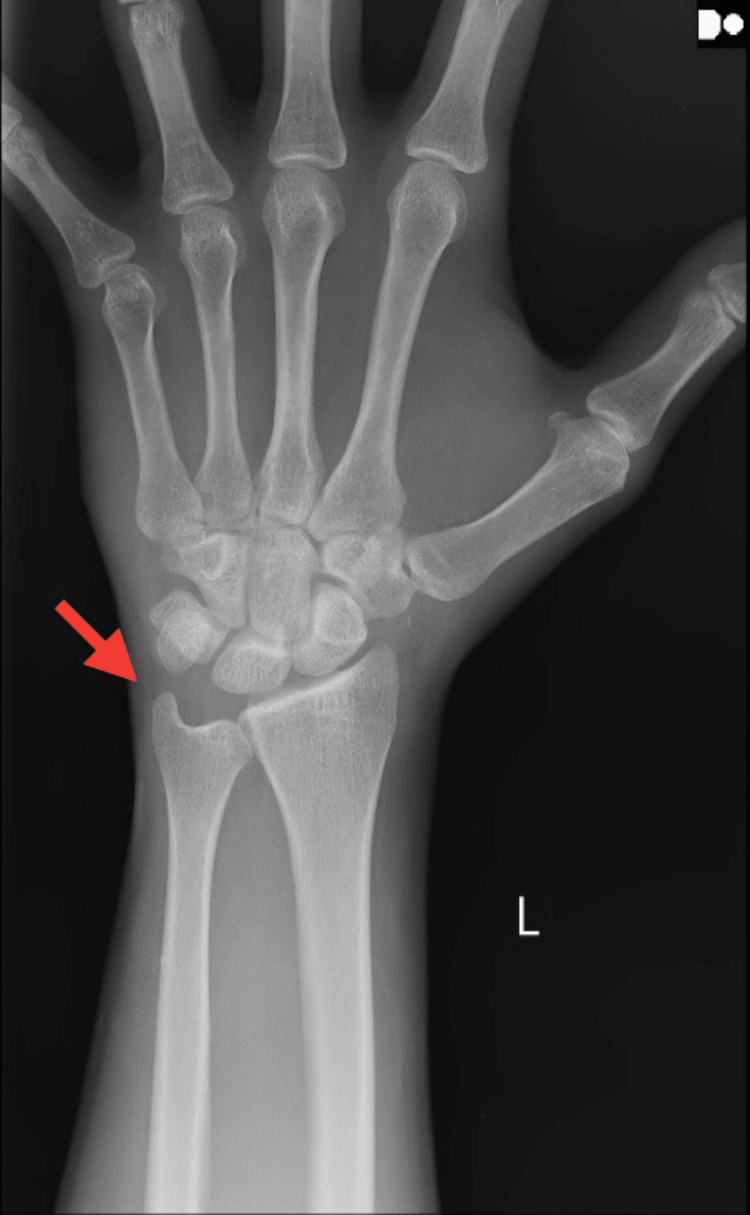
Radiograph of the left wrist in AP view showing negative ulnar variance AP, anteroposterior

**Figure 3:**
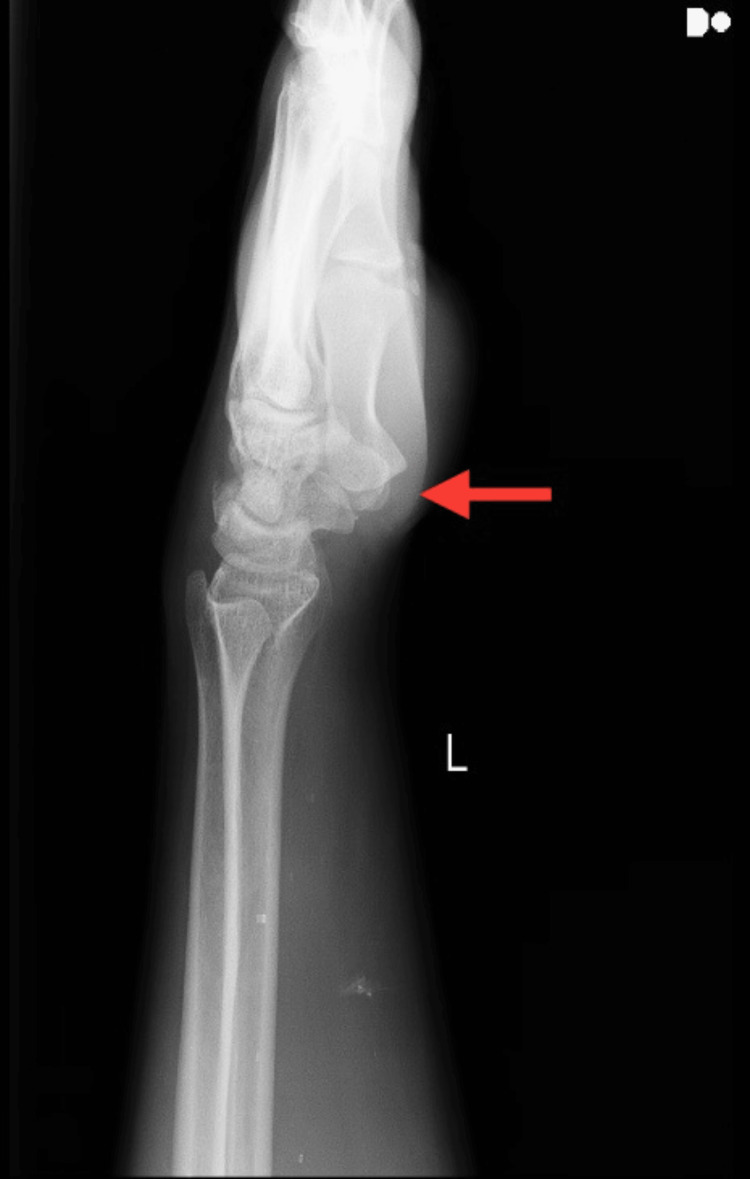
Radiograph of the left wrist in lateral view showing VISI VISI, volar intercalated segment instability

An X-ray of the left shoulder in AP view revealed a grade 1 AC joint sprain according to the Rockwood classification, as shown in Figure 1. X-rays for the left wrist in AP view revealed negative ulnar variance, as shown in Figure 2, but for the same wrist, the lateral view depicted shifting of the lunate bone on the volar aspect with the triquetrum going in a slight dorsal aspect, as shown in Figure 3, indicating VISI due to injury to the lunotriquetral ligament.

Therapeutic interventions

**Figure 4:**
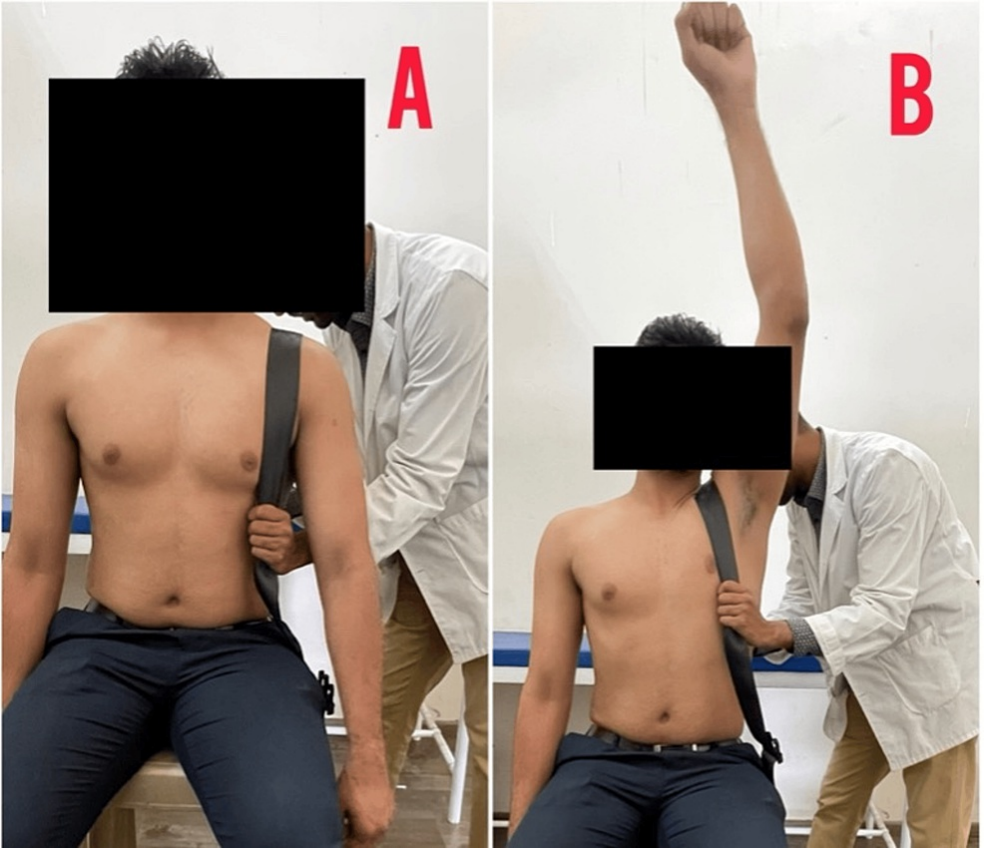
Mulligan’s movement with mobilization for the AC joint AC, acromioclavicular

**Figure 5:**
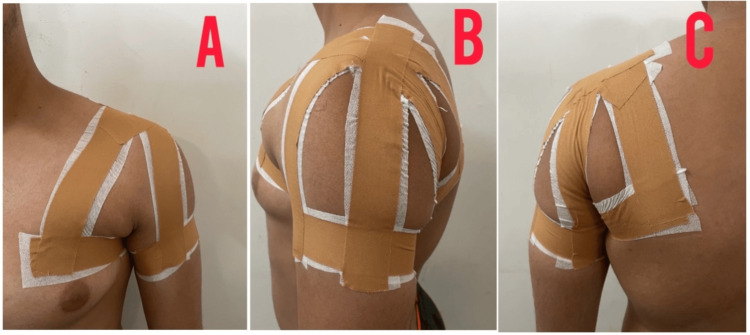
Rigid taping for the AC joint AC, acromioclavicular

**Figure 6:**
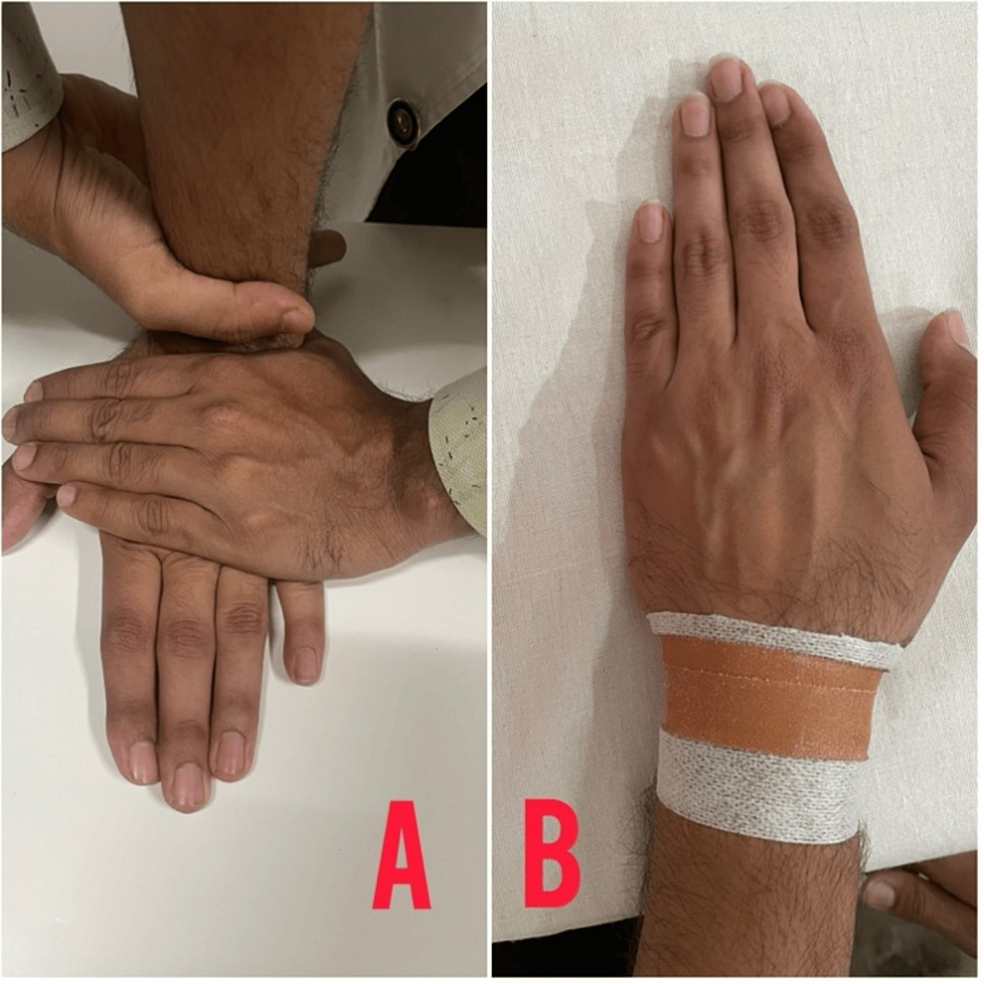
Mulligan's movement with mobilization and rigid taping for the left wrist

Table 3 depicts the rehabilitation protocol used for the patient. Figure 4 shows Mulligan’s movement with mobilization for the AC joint, Figure 5 shows a rigid taping maneuver for the correction of dysfunction, and Figure 6 shows Mulligan’s movement with mobilization for the wrist, followed by the application of rigid taping.

**Table 3:** Physiotherapeutic management implemented for the patient AC, acromioclavicular; LASER, light amplification by stimulated emission of radiation

Phases	Goals	Physiotherapy intervention	Dosage
Week 1	Patient education	Explaining the condition and the precautions to be taken associated with it:	Timely education of the patient as per the existing phase
		Avoiding lifting heavy weights with the wrist	
		Avoiding extreme overhead abduction while traveling by holding a handlebar	
	To reduce pain and inflammation	Cryotherapy: application of an ice pack to the affected areas	For 15-20 minutes, two to three times a day
		LASER therapy	For 10 minutes at 1.5 J cm^-2^
	Improving stability for:	Correction of the dysfunction by Mulligan’s movement with mobilization and reinforcement by rigid taping	For one week and changing taping every alternate day
	AC joint		
	Lunotriquetral ligament		
Week 2-3	To improve muscle strength	Isometrics for external rotators	Perform 10 repetitions for three sets and hold for 10 seconds
		Isometrics for internal rotators	Perform 10 repetitions for three sets and hold for 10 seconds
		Isometrics for abductors	Perform 10 repetitions for three sets and hold for 10 seconds
		Isometrics for wrist flexors and extensors	Perform 10 repetitions for three sets and hold for 10 seconds
Week 4-5	To improve range of motion	Pectoral and anterior deltoid stretch	Hold for 20-30 seconds and repeat three to five times
		Shoulder lateral raise, shoulder horizontal adduction, and wall climbs	Perform 15 repetitions for three sets
		Wrist flexion and extension exercises	Perform 15 repetitions of three sets
Week 6	To maintain and increase muscle strength	External rotation with a resistance band	Perform 10 repetitions for three sets
		Internal rotation with a resistance band	Perform 10 repetitions for three sets
		Abduction with a resistance band	Perform 10 repetitions for three sets
		Flexion and extension of the wrist via blue TheraBand	Perform 10 repetitions for three sets

Outcome measures

Table 4 shows the intervention outcomes for the patient.

**Table 4:** Outcomes of the intervention used for the patient DASH, Disabilities of the Arm, Shoulder, and Hand; SPADI, Shoulder Pain and Disability Index; VAS, Visual Analogue Scale

Outcomes	Week 1	Week 2-3	Week 4-5	Week 6
VAS	On rest: 4/10; on activity: 7/10	On rest: 2/10; on activity: 5/10	On rest: 2/10; on activity: 3/10	On rest: 1/10; on activity: 2/10
DASH	58%	39%	23%	12%
SPADI	60%	34%	18%	10%

## Discussion

This case underscores the significance of comprehensive assessment and management for a 22-year-old male patient who came to the outpatient department following an RTA. Subsequent clinical evaluation revealed a grade 1 AC joint sprain and VISI at the wrist. The documentation of AC joint injuries began in 1977 when Janecki published a case report involving a 19-year-old female [15]. In 2015, Satpute et al. proved in their study that the clinical effects of MWM are an effective rehabilitation intervention for managing patients with acute shoulder dysfunction; moreover, they also proved that the MWM technique is superior in comparison with other treatment options available [16]. Amro et al. found that the inclusion of MWM and therapeutic taping techniques along with traditional physiotherapeutic protocols provided better outcomes. The authors also concluded that Mulligan’s techniques improve functional activities by alleviating pain [17]. The biomechanical properties of the capsule and their effects on translational and rotational stability were extensively examined by Dyrna et al. in their research. After careful dissection of the complete capsule, while ensuring the coracoclavicular ligaments remain intact, the resistance to rotational and horizontal stress tests witnessed a substantial decline, measuring less than 25% and 10% of the original condition, respectively [18].

These findings hold significant value in understanding the essential role of the correct positioning of the AC ligaments and capsule, which is vital for acromial centering with respect to the clavicle throughout the scapulothoracic motion. Any disturbance in the stability of dynamics can lead to problems like compromised clavicular strut function, as well as glenohumeral and scapulothoracic dyskinesias [19]. In 2023, Helvey-Byers et al. showed mobilization techniques with therapeutic exercises for the shoulder complex as an effective protocol for managing grade 1 AC and sternoclavicular sprains [20]. Post-traumatic volar instability is a condition that can go unnoticed during initial traumatic instances. VISI on radiographs could be identified by analyzing multiple angles, including a loss of scapholunate angle, a reduction in congruence among lunate, capitate, and radius, and an increase in capitolunate angle [21]. Early diagnosis can result in conservative management via physical therapy rehabilitation; other options can range from closed reduction and Kirschner wire fixation to tenodesis, arthrodesis, etc. [22].

## Conclusions

This report highlights the case of a 22-year-old male patient who injured his shoulder and wrist following an RTA. His clinical assessment identified a grade 1 AC joint sprain and VISI at the wrist. A multidisciplinary conservative management approach by the medical team, along with tailored physical therapy rehabilitation, was implemented to alleviate pain, restore function, and promote optimal recovery for the patient. This case adds to the growing body of evidence supporting the effectiveness of physiotherapy interventions in the conservative management of AC joint sprains and carpal instabilities, advocating for a patient-centered approach in the rehabilitation process. Future research should focus on comparing various conservative management strategies to optimize recovery times and outcomes for similar injuries.
